# Supplementary material for: Effectiveness of early versus delayed rehabilitation following rotator cuff repair: Systematic review and meta-analyses
Source: PLoS One. 2021 May 28;16(5):e0252137. doi: 10.1371/journal.pone.0252137 (PMC8162656; doi:10.1371/journal.pone.0252137)
Supplement: S1 File — (DOCX) [file pone.0252137.s001.docx]

**S1 File.** Search strategy

1. Shoulder/ or Shoulder Injuries/ or Shoulder Joint/ or Shoulder Pain/

2. Rotator Cuff/

3. "Physical and Rehabilitation Medicine"/ or Rehabilitation/

4. Physical Therapy Modalities/ or Exercise Therapy/

5. Physiotherapy.mp. [mp=title, abstract, original title, name of substance word, subject heading word, floating sub-heading word, keyword heading word, organism supplementary concept word, protocol supplementary concept word, rare disease supplementary concept word, unique identifier, synonyms]

6. 1 or 2

7. 3 or 4 or 5

8. 6 and 7

9. randomi*ed controlled trial.mp. [mp=title, abstract, original title, name of substance word, subject heading word, floating sub-heading word, keyword heading word, organism supplementary concept word, protocol supplementary concept word, rare disease supplementary concept word, unique identifier, synonyms]

10. Clinical Trial/ or Controlled Clinical Trial/ or Pragmatic Clinical Trial/

11. 9 or 10

12. 8 and 11
