# Supplementary material for: Effectiveness of early versus delayed rehabilitation following rotator cuff repair: Systematic review and meta-analyses
Source: PLoS One. 2021 May 28;16(5):e0252137. doi: 10.1371/journal.pone.0252137 (PMC8162656; doi:10.1371/journal.pone.0252137)
Supplement: S2 File — (DOCX) [file pone.0252137.s002.docx]

**S2 File.** Characteristics of the included RCTs.

| **Author (year)** | **Country** | **No. of patients randomised**  **E/D – M/F** | **Age**  **(years) E/D** | **Tear characteristics** | **Surgery characteristics** | **Outcomes** |
| --- | --- | --- | --- | --- | --- | --- |
| Arndt, Clavert (1) | France | 49/43 – 58/34 | 55.3 | Non-retracted isolated tears of supraspinatus; partial-thickness: 24%, full-thickness: 76% | 5 surgeons;  59% single row, 41% double row; LHB tenotomy: 65%, LHB tenodesys:11%; acromioplasty: 91% | CM, cuff integrity (arthrogram, CT or arthro-MRI), ROM |
| Cuff and Pupello (2) | USA | 33/35 – 38/30 | 63/63.5 | Supraspinatus; full-thickness; crescent-shaped | Transosseous suture bridge | ASES, cuff integrity (US), ROM, SST |
| De Roo, Muermans (3) | Belgium | 79/51 – 89/41 | 65.1/64.6 | Small to large; full-thickness | Single or double row; acromioplasty | CM, cuff integrity (US) ROM, SPADI, SST, strength (dynamometer), UCLA |
| Duzgun, Baltaci (4) | Turkey | 13/16 – 3/26 | 55.8/56.6 | Medium and large | NA | DASH, pain (VAS), ROM |

***Continue***

**Supplementary file 2 (continue).** Characteristics of the included RCTs.

| **Author (year)** | **Country** | **No. of patients randomised**  **E/D – M/F** | **Age**  **(years) E/D** | **Tear characteristics** | **Surgery characteristics** | **Outcomes** |
| --- | --- | --- | --- | --- | --- | --- |
| Duzgun, Baltaci (5) | Turkey | 20/22 – 6/34 | 57.6/57.2 | Medium and large | NA | ROM |
| Fawzy, Rizk Mohamed (6) | Egypt | 86/86 – 90/74 | 57.8/57 | Small to Medium sized; full-thickness | Single-row and subacromial decompression; no biceps procedures | ASES, CM, cuff integrity (MRI), pain (VAS) ROM |
| Jenssen, Lundgreen (7) | Norway | 60/60 – 69/49 | 56/55 | Supraspinatus or upper infraspinatus; small to medium sized; full-thickness | Different surgeons; single-row using 1 or 2 triple-loaded suture anchors; subacromial decompression; LHB tenotomy/tenodesis (no difference between groups ); ACJ resections (E: 8% vs. D: 23%) | CM, cuff integrity (MRI), ROM, WORC |
| Keener, Galatz (8) | USA | 67/62 – 73/51 | 55.8/54.8 | Supraspinatus and/or infraspinatus; Small and medium; full-thickness | 3 surgeons; double row transosseous; acromioplasty; LHB tenodesis or tenotomy | ASES, CM, cuff integrity (US), pain (VAS) ROM, SST, strength |

***Continue***

**Supplementary file 2.** Characteristics of the included RCTs.

| **Author (year)** | **Country** | **No. of patients randomised**  **E/D – M/F** | **Age**  **(years) E/D** | **Tear characteristics** | **Surgery characteristics** | **Outcomes** |
| --- | --- | --- | --- | --- | --- | --- |
| Kim, Chung (9) | Korea | 60/57 - 44/61 | 60/60 | Small and medium; full-thickness | Different surgeons; single row: 17, double row: 2, suture bridge: 86; acromioplasty | ASES, CM, cuff integrity (US,MRI or CT), pain (VAS) ROM, SST |
| Kjær (10) | Denmark | 41/41 – 64/28 | 59/61 | Supraspinatus involved: E: 100% vs D:100%  Infraspinatus involved: E:26% vs D:36%  Subscapularis involved: E:7.3% vs D:17.1% | NA | Cuff integrity (US), DASH, pain (NRS), ROM, strength (dynamometer), WORC |
| Klintberg, Gunnarsson (11) | Sweden | 9/9 – 9/5 | 55 | Full-thickness | NA | CM, FIS, pain (VAS) ROM, strength (isokinetics) |
| ***Continue*** |  |  |  |  |  |  |

**Supplementary file 2.** Characteristics of the included RCTs.

| **Author (year)** | **Country** | **No. of patients randomised**  **E/D – M/F** | **Age**  **(years) E/D** | **Tear characteristics** | **Surgery characteristics** | **Outcomes** |
| --- | --- | --- | --- | --- | --- | --- |
| Koh, Lim (12) | Korea | 47/53 – 44/44 | 60.1/59.5 | Postero-superior; medium; full-thickness;  2-4 cm | Single row, acromioplasty, capsular release | ASES, CM, cuff integrity (MRI), VAS |
| Lee, Cho (13) | Korea | 43/42 – 41/23 | 54.5/55.2 | Medium: 41, large: 45; full-thickness | One surgeon; single row; patients who need LHB, acromion and/or clavicle procedures were excluded | Cuff integrity (MRI), ROM, strength (dynamometer), UCLA, VAS |
| Littlewood, Bateman (14) | UK | 37/36 – 42/31 | 60.6/65.4 | All sizes included: E:2.96 cm vs D:2.5 cm  Supraspinatus involved: E: 28 vs D:30  Infraspinatus involved: E:7 vs D:6  Subscapularis involved: E:1% vs D:6% | Eight surgeons;  complete repairs: E: 17 vs D:30  partial repairs:  E: 6 vs D:0 | Cuff integrity (US), EQ-5D-5L, OSS, SPADI |
| ***Continue*** |  |  |  |  |  |  |

**Supplementary file 2.** Characteristics of the included RCTs.

| **Author (year)** | **Country** | **No. of patients randomised**  **E/D – M/F** | **Age**  **(years) E/D** | **Tear characteristics** | **Surgery characteristics** | **Outcomes** |
| --- | --- | --- | --- | --- | --- | --- |
| Mazzocca, Arciero (15) | USA | 36/37 – 40/18 | 54/55 | Supraspinatus; full-thickness | Single surgeon; transosseous equivalent; three to four anchors; subacromial decompression; LHB tenodesis | ASES, CM, cuff integrity (MRI), ROM, SANE, SST, WORC |
| Oyarzún, Poblete (16) | Chile | 15/15 – 22/8 | NA | Supraspinatus | NA | Pain (VAS), ROM |
| Raschhofer, Poulios (17) | Austria | 14/16 – 19/10 | 56.3/59.5 | Medium sized; full-thickness | Single-row; subacromial decompression; biceps tenotomy and ACJ resection | CM, DASH, pain (VAS), ROM, strength (dynamometer) |
| ***Continue*** |  |  |  |  |  |  |

**Supplementary file 2.** Characteristics of the included RCTs.

| **Author (year)** | **Country** | **No. of patients randomised**  **E/D – M/F** | **Age**  **(years) E/D** | **Tear characteristics** | **Surgery characteristics** | **Outcomes** |
| --- | --- | --- | --- | --- | --- | --- |
| Sheps, Bouliane (18) | Canada | 97/92 – 115/74 | 55.4/54.9 | All tear sizes included.  Single tendon involvement: E:79% vs D: 84%; tear sizes: Small: E:28% vs D: 26%  Medium: E: 51% vs D: 56%  Large: E: 21% vs D:18%;  full-thickness | Multiple surgeons; mini-open method | Cuff integrity (NA), pain (VAS), ROM, strength (tensiometer), WORC |
| ***Continue*** |  |  |  |  |  |  |

**Supplementary file 2.** Characteristics of the included RCTs.

| **Author (year)** | **Country** | **No. of patients randomised**  **E/D – M/F** | **Age**  **(years) E/D** | **Tear characteristics** | **Surgery characteristics** | **Outcomes** |
| --- | --- | --- | --- | --- | --- | --- |
| Sheps, Silveira (19) | Canada | 103/103 – 131/75 | 55.5/56.2 | All tear sizes included.  Mean length of tear AP: E: 2.1 cm vs D: 2.1 cm  Mean length of tear ML: E: 1.9 cm vs D: 1.9 cm | Multiple surgeons; arthroscopic method. Single row: E: 10.7% vs D:10.7%  Double row/transosseous: E: 89.3% vs D: 89.3%  Tenodesis: E: 45% vs D: 44.1  Acromioplasty: E: 75.7% vs D:77.5%  ACJ excision: E:15.5% vs D:9.7% | Cuff integrity (US), pain (VAS), ROM, SF-36, strength, (dynamometer), WORC |
| Tirefort, Schwitzguebel (20) | Switzerland | 40/40 – 37/43 | 54.7/53.5 | Isolated superior full thickness tear; small to medium sized | Double row suture anchors.  Tenodesis: E:53% vs D: 65%  Tenotomy: E: 38% vs D: 33%  Acromioplasty: E:98% vs D: 88% ACJ resection: E:35% vs D: 33% | ASES, cuff integrity (US), pain (VAS), ROM, SANE |

ACJ: acromioclavicular joint, AP: anteroposterior, ASES: American Shoulder and Elbow Surgeons, CT: Computed Tomography, CM: Constant-Murley Score, E/D: Early/Delayed, FIS: Functional Index of the Shoulder, LHB: Long Head of Biceps, MRI: Magnetic Resonance Imaging, M/F: Male/Female, ML: mediolateral, NA: Not Available, OSS: Oxford Shoulder Score, ROM: Range Of Motion, RCT: Rotator Cuff Tear, SANE: Single Assessment Numeric Evaluation score, SPADI: Shoulder Pain and Disability Index, SST: Simple Shoulder Test Score, US: Ultrasound, UCLA: University of California Los Angeles, VAS: Visual Analogue Scale, WORC: Western Ontario Rotator Cuff index.
