# Supplementary material for: Effectiveness of early versus delayed rehabilitation following rotator cuff repair: Systematic review and meta-analyses
Source: PLoS One. 2021 May 28;16(5):e0252137. doi: 10.1371/journal.pone.0252137 (PMC8162656; doi:10.1371/journal.pone.0252137)
Supplement: S3 File — (DOCX) [file pone.0252137.s003.docx]

**S3 File.** Risk of bias by RCT.
