# Supplementary material for: Effectiveness of early versus delayed rehabilitation following rotator cuff repair: Systematic review and meta-analyses
Source: PLoS One. 2021 May 28;16(5):e0252137. doi: 10.1371/journal.pone.0252137 (PMC8162656; doi:10.1371/journal.pone.0252137)
Supplement: S4 File — (DOCX) [file pone.0252137.s004.docx]

**S4 File.** Characteristics of the rehabilitation programmes.

| **Author (year)** | **Early Rehabilitation** | **Delayed Rehabilitation** |
| --- | --- | --- |
| Arndt, Clavert (1) | **IP:** Sling for 6 weeks  **First day postoperative-week 6:** Pendulum exercise **+** manual shoulder PROM exercises **+** CPM (3-5x per week)  **Week 6-4 Months:** Shoulder AROM exercises  **From 4 months:** Strengthening exercises | **IP:** Sling for 6 weeks  **Week 0-6:** Immobilisation **+** Pendulum exercise  **Week 6-4 Months:** Shoulder AROM exercises  **From 4 months:** Strengthening exercises |
| Cuff and Pupello (2) | **IP:** Shoulder immobiliser for 6 weeks  **Weeks 0-3:** Started in the second day post-surgery. Pendulum exercise **+** shoulder PROM exercises for flexion and external rotation **+** elbow, wrist and hand AROM exercises  **Weeks 4-6:** Similar to week 0-3 **+** progressing ROM **+** AROM elbow, wrist and hand  **Weeks 6-10:** Shoulder AAROM exercises  **Weeks 10-12:** Shoulder AAROM **+** AROM exercises  **From week 12:** Strengthening  Face-to-face sessions 3x per week | **IP:** Shoulder immobiliser for 6 weeks  **Weeks 0-3:** Pendulum exercise 3x daily for 5 minutes **+** elbow, wrist and hand AROM exercises  **Weeks 4-6:** Pendulum exercise 3x daily for 5 minutes **+** elbow, wrist and hand AROM exercises  **Weeks 6-10:** Shoulder PROM exercises **+ week 7** Shoulder AROM exercises 1x per week  **Weeks 10-12:** Shoulder AAROM **+** AROM exercises  **From week 12:** Strengthening |
| ***Continue*** |  |  |

**Supplementary file 4.** Characteristics of the rehabilitation programmes.

| **Author (year)** | **Early Rehabilitation** | **Delayed Rehabilitation** |
| --- | --- | --- |
| De Roo, Muermans (3) | **IP:** Brace with abduction pillow (30°) for 4 weeks during day and night **+** 2 more weeks only at night  **First day postoperative – week 5:** Pendulum exercise (3x per day, max 10 minutes, 20 cm diameter) **+** shoulder PROM for flexion, abduction, internal and external rotation **+** scapular mobilization (5 days pw)  **Weeks 5-8:** Specific capsular glenohumeral exercises **+** shoulder AAROM exercises  **From week 8:** Strengthening | **IP:** Brace with abduction pillow (30°) for 4 weeks during day and night **+** 2 more weeks only at night  **Weeks 1-4:** Pendulum exercise (3x per day, max 10 minutes, 20 cm diameter)  **Weeks 5:** Gradual shoulder PROM mobilization  **From week 6:** Similar to early mobilisation group; no further details available |
| ***Continue*** |  |  |

**Supplementary file 4.** Characteristics of the rehabilitation programmes.

| **Author (year)** | **Early Rehabilitation** | **Delayed Rehabilitation** |
| --- | --- | --- |
| Duzgun, Baltaci (4) | **IP:** NA  **Weeks 0-1:** Cold pack every 2 hours for 20 min  **Weeks 1-2:** Cold pack **+** deltoid and biceps soft-tissue mobilisation **+** shoulder PROM exercises for flexion and abduction **+** elbow and neck AROM **+** hand strengthening  **Weeks 2-3:** Cold pack **+** shoulder PROM exercises for flexion **+** elbow and neck AROM exercises **+** glenohumeral mobilization  **Weeks 3-4:** Cold pack **+** scapular mobilization **+** Shoulder AROM exercises for flexion, internal rotation, abduction **+** strengthening for biceps, triceps and serratus anterior using rubber bands  **Weeks 4-5:** Cold pack **+** shoulder AROM exercises for flexion **+** strengthening of shoulder abduction, internal rotation, external rotation with rubber bands  **Weeks 5-6:** Cold pack **+** progression of shoulder strengthening exercises with more resistant rubber bands **+** posterior capsule stretching  **Weeks 6:** Week 5-6 **+** Resistive PNF exercises  **Weeks 7:** Wall shoulder push-up **+** On-the-table press-up **+** on-the-table push-up | **IP:** NA  **Weeks 0-4:** Early rehabilitation weeks 0-1  **Weeks 4-6:** Early rehabilitation weeks 2-3  **Weeks 6-8:** Early rehabilitation weeks 3-4  **Weeks 8-10:** Early rehabilitation weeks 4-5  **Weeks 10-14:** Early rehabilitation weeks 5-6  **Weeks 14-18:** Early rehabilitation weeks 6  **Weeks 18-22:** Early rehabilitation weeks 7 |
| ***Continue*** |  |  |

**Supplementary file 4.** Characteristics of the rehabilitation programmes.

| **Author (year)** | **Early Rehabilitation** | **Delayed Rehabilitation** |
| --- | --- | --- |
| Duzgun, Baltaci (5) | **IP:** 2 weeks  **Weeks 2-7:** Soft tissue mobilization for the scapulothoracic and glenohumeral joints along with mobilisation exercises (3x week during all weeks).  **Weeks 3:** Shoulder AROM exercises for scaption, flexion and abduction  **Weeks 4:** Light resistive exercises with rubber bands. | **IP:** 4 weeks  **Weeks 4-17:** Soft tissue mobilization for the scapulothoracic and glenohumeral joints along with mobilisation exercises (3x week during all weeks).  **Weeks 6:** Shoulder AROM exercises for scaption, flexion and abduction.  **Weeks 8:** Light resistive exercises with rubber bands. |
| Fawzy, Rizk Mohamed (6) | **IP:** Sling for 6 weeks  **Day 2:** Shoulder PROM exercises for flexion and external rotation + pendulum exercise + elbow, wrist and hand AROM exercises  **Week 6:** Shoulder AROM exercises  **Weeks 12:** Shoulder AROM exercises and strengthening exercises  **From 6 months:** Sports activities  Face-to-face sessions 3x week | **IP:** Sling for 6 weeks  **Week 6:** Shoulder PROM exercises for flexion and external rotation + pendulum + elbow, wrist and hand AROM exercises  **Week 9:** Shoulder AROM exercises  **Weeks 12:** Shoulder AROM exercises + shoulder strengthening exercises  **From 6 months:** Sports activities  Face-to-face sessions 3x week |
| ***Continue*** |  |  |

**Supplementary file 4.** Characteristics of the rehabilitation programmes.

| **Author (year)** | **Early Rehabilitation** | **Delayed Rehabilitation** |
| --- | --- | --- |
| Jenssen, Lundgreen (7) | **IP:** Simple sling for 3 weeks  **Day 1:** Elbow and hand AROM exercises + shoulder PROM exercises + pendulum exercises  **Week 3:** Shoulder AROM exercises (lifting anything greater than the weight of the arm for the first 3 months).  **From 6 months:** Heavy lifting and weight training  Face-to-face sessions 2-3x a week | **IP:** Brace with a small abduction pillow for 6 weeks  **Day 1:** Elbow and hand AROM exercises + shoulder PROM exercises + pendulum exercises  **Week 6:** Shoulder AROM exercises (lifting anything greater than the weight of the arm for the first 3 months).  **From 6 months:** Heavy lifting and weight training  Face-to-face sessions 2-3x a week |
| Keener, Galatz (8) | **IP:** Sling for 6 weeks  **Immediate postoperative:** Pendulum exercise **+** elbow, wrist and hand AROM exercises  **Weeks 1-6:** Shoulder PROM performed by therapist  **Weeks 6-12:** Shoulder AAROM and AROM exercises  **3-4 Months:** Deltoid and scapular stabilizer strengthening  **From 4 months:** Full activities based on patient's progress | **IP:** Sling for 6 weeks  **Immediate postoperative – week 6:** Elbow, wrist and hand AROM exercises; no shoulder mobilization  **Week 6-12:** Shoulder PROM performed by therapist  **3-4 Months:** Shoulder AAROM and AROM exercises  **From 4 months:** Deltoid and scapular stabilizer strengthening; full activities between 5 and 6 months based on patients progress |
| ***Continue*** |  |  |

**Supplementary file 4.** Characteristics of the rehabilitation programmes.

| **Author (year)** | **Early Rehabilitation** | **Delayed Rehabilitation** |
| --- | --- | --- |
| Kim, Chung (9) | **IP:** Brace with abduction pillow (30°) during 4 or 5 weeks  **First day postoperative- week 4/5:** Shoulder PROM exercises for flexion, abduction and external rotation **+** elbow, wrist and hand AROM exercises **+** shrugging of shoulders  **Week 4/5:** Shoulder AAROM exercises  **Week 9/12:** Muscle strengthening  **6 Months:** Return of activities | **IP:** Brace with abduction pillow (30°) during 4 or 5 weeks  **First day postoperative- week 4/5:** Elbow, wrist and hand AROM exercises **+** shrugging of shoulders  **Week 4/5:** Shoulder AROM exercises  **Week 9/12:** Muscle strengthening  **6 Months:** Return of activities |
| Kjær (10) | **IP:** 2 weeks in fixed sling followed by 3 weeks in standard sling  **Weeks 2-5:** Physiotherapist guided PROM exercises + close-chain AAROM and AROM exercises (week 2 only) - AAROM and AROM Restrictions: ABD + FLEX < 90 degrees; IR < 90 degrees in neutral; ER = 0 degrees in neutral  **Weeks 3-5:** Close-chain AAROM and AROM exercises - AAROM and AROM Restrictions: ABD + FLEX < 90 degrees; IR < 90 degrees in neutral; ER < 45 degrees in neutral  **Weeks 6-12:** Therapist-supervised AROM (FLEX, ABD, EXT, ER and IR) with gradually (individually) increased loading and progression from close-chain to open-chain exercises  **Weeks 12-20:** Continuation of rehabilitation in the community  Face-to-face sessions 3x a week | **IP:** 2 weeks in fixed sling followed by 3 weeks in standard sling  **Weeks 2-5:** Physiotherapist guided PROM exercises PROM Restrictions: ABD + FLEX: None IR < 90 degrees in neutral ER < 45 degrees in neutral  **Weeks 6-12:** Therapist-supervised AROM (FLEX, ABD, EXT, ER and IR) with gradually (individually) increased loading and progression from close-chain to open-chain exercises  **Weeks 12-20:** Continuation of rehabilitation in the community  Face-to-face sessions 1x a week |

***Continue***

**Supplementary file 4.** Characteristics of the rehabilitation programmes.

| **Author (year)** | **Early Rehabilitation** | **Delayed Rehabilitation** |
| --- | --- | --- |
| Klintberg, Gunnarsson (11) | **IP:** Sling for 4 weeks  **First day to 4 weeks:** Activation of the rotator cuff + shoulder PROM exercises  **Weeks 4-6 weeks:** Increased loading of the rotator cuff + shoulder AAROM exercises (including in the pool)  **Weeks 6-8:** Shoulder AROM exercises + PROM exercise for shoulder internal rotation  **Weeks 8-10:** Resisted exercises for the rotator cuff + dynamic strengthening exercises for the rotator cuff + scapular muscles through range  **Weeks 10-12:** Water-resisted exercises; eccentric loading of the rotator cuff  **Weeks 12-16:** Eccentric load on the rotator cuff during supervised physiotherapy  **Weeks 16-24:** Dynamic strengthening for rotator cuff and scapular muscles throughout full range + active, water-resisted exercises performed throughout full ROM  **From 24 weeks:** Eccentric load on the rotator cuff during supervised physiotherapy | **IP:** Sling for 6 weeks  **First day to 4 weeks:** Shoulder PROM exercises  **Weeks 6-10:** Shoulder AAROM exercises (at six weeks activation of the rotator cuff)  **Weeks 10-16:** Aquatic AAROM shoulder exercises  **Weeks 16-24:** Strengthening water-resisted exercises + eccentric loading of the rotator cuff  **From 24 weeks:** Eccentric load on the rotator cuff during supervised physiotherapy |
| ***Continue*** |  |  |

**Supplementary file 4.** Characteristics of the rehabilitation programmes.

| **Author (year)** | **Early Rehabilitation** | **Delayed Rehabilitation** |
| --- | --- | --- |
| Koh, Lim (12) | **IP:** Sling with an abduction pillow (20°) during 4 weeks  **Week 5-10:** Shoulder PROM exercises with rope, pulley and cane **+** home-based exercise.  Shoulder AAROM and AROM were allowed as patients obtained nearly full PROM  **Week 11- 6 Months:** Shoulder strengthening using elastic bands  **6 Months:** Return to normal activities, including sports | **IP:** Sling with an abduction pillow (20°) during 8 weeks  **Week 9-14:** Shoulder PROM exercises, pulley and cane **+** home-based exercise. Shoulder AAROM and AROM were allowed as patients obtained nearly full PROM  **Week 15 – 6 Months:** Shoulder strengthening using elastic bands  **6 Months:** Return to normal activities, including sports |
| Lee, Cho (13) | **IP:** Sling with an abduction pillow (30°) during 6 weeks  **First day postoperative – week 6:** Shoulder PROM exercises for flexion and external rotation (2x pd) **+** pendulum exercises **+** shoulder PROM exercises (3x pd) **+** home-based exercises  **Week 6-on:** Shoulder AROM **+** PROM exercises for all directions | **IP:** Sling with an abduction pillow (30°) during 6 weeks  **First day postoperative – week 3:** Self-PROM shoulder flexion **+** CPM (2x pd)  **Week 3-6:** Shoulder PROM exercises (2x pd)  **Week 6-on:** Shoulder strengthening using elastic bands |
| ***Continue*** |  |  |

**Supplementary file 4.** Characteristics of the rehabilitation programmes.

| **Author (year)** | **Early Rehabilitation** | **Delayed Rehabilitation** |
| --- | --- | --- |
| Littlewood, Bateman (14) | **IP:** Advice to remove the sling as soon as possible  **From week 0:** Advice to patients gradually begin to active use of their arms as soon as able within acceptable limits of pain.  One face-to-face session with physiotherapist before hospital discharge and at 2 weeks postoperative.  **After 4 weeks:**  Individualised progression between phases  Further 4 face-to-face sessions  **Phase 1:** Progress to assisted movement then full active movement within pain limits  **Phase 2:** Isometric exercises for all shoulder muscle groups  **Phase 3:** Resisted exercises through range, within limits of pain  **Phase 4:** Functional restoration | **IP:** Sling for 4 weeks  **From week 0:** table slides + elbow, wrist and hand exercises AROM exercises + shoulder PROM exercises for abduction, flexion and lateral rotation movements, within pain limits  One face-to-face session with physiotherapist before hospital discharge and at 2 weeks postoperative.  **After 4 weeks:**  Individualised progression between phases  Further 4 face-to-face sessions  **Phase 1:** Progress to assisted movement then full active movement within pain limits  **Phase 2:** Isometric exercises for all shoulder muscle groups  **Phase 3:** Resisted exercises through range, within limits of pain  **Phase 4:** Functional restoration |
| ***Continue*** |  |  |

**Supplementary file 4.** Characteristics of the rehabilitation programmes.

| **Author (year)** | **Early Rehabilitation** | **Delayed Rehabilitation** |
| --- | --- | --- |
| Mazzocca, Arciero (15) | **IP:** Ultrasling with abduction pillow for 6 weeks  **Day 2 or 3 to week 6:** Shoulder AAROM exercises (external rotation & flexion)  **Week 7:** Shoulder AAROM exercises + stretching  **Week 8-12:** Shoulder AROM exercise + stretching  **Week 13:** Shoulder isometric strengthening  **Week 14:** Shoulder strengthening using theraband for external and internal rotation  **Week 15:** Strengthening using theraband for external and internal rotation, and abduction  **From week 16:** Strengthening using theraband for external and internal rotation, and abduction + PNF  Face-to-face session 2x week | **IP:** Ultrasling with abduction pillow for 6 weeks  **Week 5:** Shoulder AAROM exercises (external rotation & flexion)  **Week 7:** Shoulder AAROM exercises + stretching  **Week 8-12:** Shoulder AROM exercise + stretching  **Week 13:** Isometric strengthening  **Week 14:** Strengthening using theraband for external and internal rotation  **Week 15:** Strengthening using theraband for external and internal rotation, and abduction  **From week 16:** Strengthening using theraband for external and internal rotation, and abduction + PNF  Face-to-face session 2x week |
| Oyarzún, Poblete (16) | **IP:** Sling + abduction component for 1 week  **Weeks 2-4:** Shoulder PROM exercises for flexion & external rotation + shoulder AROM exercise for adduction and trunk rotations + isometric strengthening for shoulder internal rotation and hand gripping + strengthening exercises for elbow flexion  Face-to-face sessions 4x week for 4 weeks | **IP:** Sling + abduction component for 4 weeks  **Weeks 2-4:** Pendulum exercise 2x day for 4x week |
| ***Continue*** |  |  |

**Supplementary file 4.** Characteristics of the rehabilitation programmes.

| **Author (year)** | **Early Rehabilitation** | **Delayed Rehabilitation** |
| --- | --- | --- |
| Raschhofer, Poulios (17) | **IP:** Sling for 6 weeks  **Week 1:** Elbow and shoulder girdle AROM exercises + shoulder PROM exercise elevation (45°) and abduction (45°)  **Weeks 2-6:** Isometric strengthening  **Week 6-onwards:** dynamic activation of the rotator cuff and strengthening of shoulder muscles  20 sessions in 12 weeks | **IP:** Sling for 6 weeks  **Week 1:** Elbow and shoulder girdle AROM exercises + shoulder PROM exercise elevation (45°) and abduction (45°)  **Weeks 2-6:** no information  **Week 6-onwards:** dynamic activation of the rotator cuff and strengthening of shoulder muscles  20 sessions in 12 weeks |
| Sheps, Bouliane (18) | **IP:** Sling as needed  Shoulder PROM, AAROM and AROM exercises for ADLs as early as pain allowed.  After 6 weeks the rehabilitation programme was identical between groups. No further information was available for the rehabilitation programme from 6 weeks postoperative | **IP:** Sling for 6 weeks Shoulder PROM and AAROM were allowed No active movements of the shoulder. After 6 weeks the rehabilitation programme was identical between groups. No further information was available for the rehabilitation programme from 6 weeks postoperative |
| ***Continue*** |  |  |

**Supplementary file 4 (continue).** Characteristics of the rehabilitation programmes.

| **Author (year)** | **Early Rehabilitation** | **Delayed Rehabilitation** |
| --- | --- | --- |
| Sheps, Silveira (19) | **IP:** Sling only for comfort  **Weeks 0-6:** Pendulum exercises + shoulder AAROM as pain allows + elbow and hand AROM exercises + pain-free AROM  To begin general conditioning program of choice  **Weeks 6-10:** Shoulder AROM exercises as pain allows – all planes + gentle stretching into terminal ROM + initiate closed chain exercises.  **Weeks 10-26:** Isometric strengthening; progress to isotonic strengthening within pain-free ROM  Closed chain strengthening  Overhead strengthening once full ROM achieved and pain well controlled.  Should not lift >15 lb. unless specified by physician  Continue with stretching (therapist may now assist)  Joint mobilization permitted | **IP:** Sling for 6 weeks **Weeks 0-6:** Pendulum exercises + shoulder AAROM were allowed + elbow and hand AROM exercises No active movements of the shoulder  To begin general conditioning program of choice  **Weeks 6 -10:** Shoulder AROM exercises as pain allows – all planes + gentle stretching into terminal ROM + initiate closed chain exercises.  **Weeks 10-26:** Isometric strengthening; progress to isotonic strengthening within pain-free ROM  Closed chain strengthening  Overhead strengthening once full ROM achieved and pain well controlled.  Should not lift >15 lb. unless specified by physician  Continue with stretching (therapist may now assist)  Joint mobilization permitted |
| ***Continue*** |  |  |

**Supplementary file 4 (continue).** Characteristics of the rehabilitation programmes.

| **Author (year)** | **Early Rehabilitation** | **Delayed Rehabilitation** |
| --- | --- | --- |
| Tirefort, Schwitzguebel (20) | **IP:** No sling  **Week 0-4:** Shoulder PROM and AAROM exercises  **From 4 weeks:** Progressive shoulder AROM exercises with elbow at the side. No exercises involving lifting of the elbow in any direction, unless assisted, were permitted  **From 8 weeks:** Demanding activities and light sports  **From 12 weeks:** Shoulder strengthening | **IP:** Sling for 4 weeks  **Week 0-4:** Shoulder PROM and AAROM exercises  **From 4 weeks:** Progressive shoulder AROM exercises with elbow at the side. No exercises involving lifting of the elbow in any direction, unless assisted, were permitted  **From 8 weeks:** Demanding activities and light sports  **From 12 weeks:** Shoulder strengthening |

AAROM: Active Assisted Range of movement, ABD: Abduction, ADLs: Activities of Daily Living, CPM: Continuous Passive Motion, ER: External Rotation, FLEX: Flexion, GH: glenohumeral, IP: Immobilisation Period, IR: Internal Rotation, NA: Not Available, PNF: Proprioceptive neuromuscular facilitation, PROM: Passive Range of Movement, ROM: Range Of Movement.
