# Supplementary material for: Effectiveness of early versus delayed rehabilitation following rotator cuff repair: Systematic review and meta-analyses
Source: PLoS One. 2021 May 28;16(5):e0252137. doi: 10.1371/journal.pone.0252137 (PMC8162656; doi:10.1371/journal.pone.0252137)
Supplement: S5 File — (DOCX) [file pone.0252137.s005.docx]

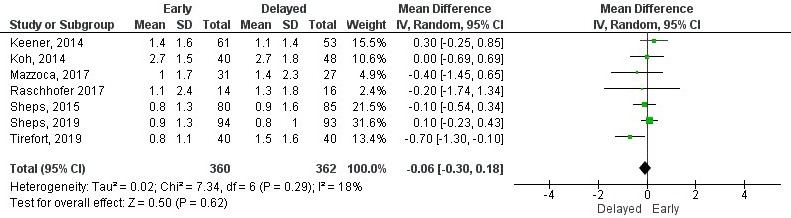
**S5 File.** Forest plots.

**Supplementary file 5.1**. Forest plot of pain intensity at six months by visual analogue scale.


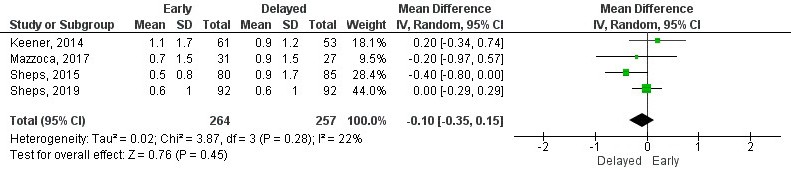


**Supplementary file 5.2**. Forest plot of pain intensity at one year by visual analogue scale.


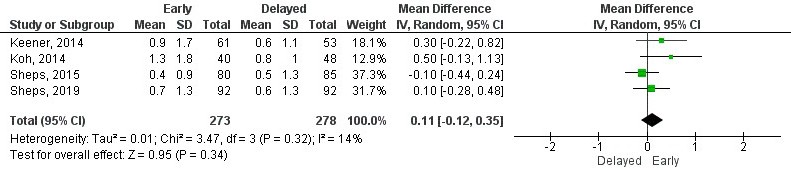
**Supplementary file 5.3**. Forest plot of pain intensity at two years by visual analogue scale.


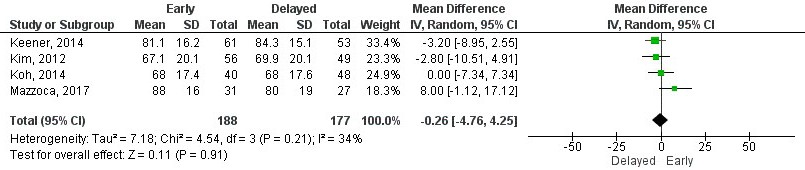


**Supplementary file 5.4**. Forest plot of function at six months by American Shoulder and Elbow Surgery score.


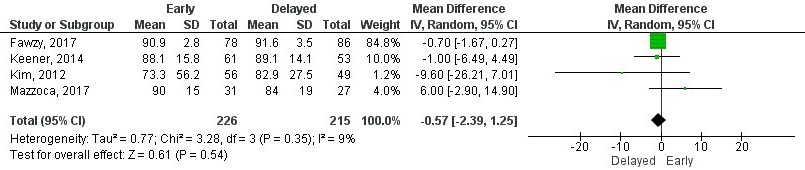
**Supplementary file 5.5**. Forest plot of function at one year by American Shoulder and Elbow Surgery score.


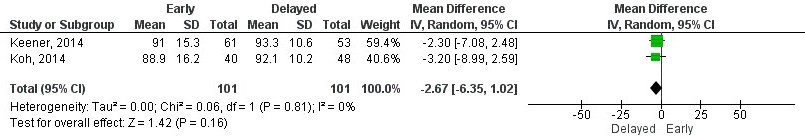


**Supplementary file 5.6**. Forest plot of function at two years by American Shoulder and Elbow Surgery score.


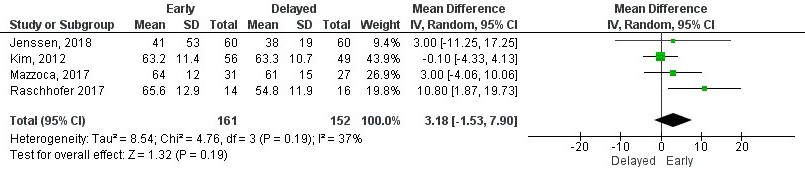
**Supplementary file 5.7**. Forest plot of function at three months years by Constant-Murley score.


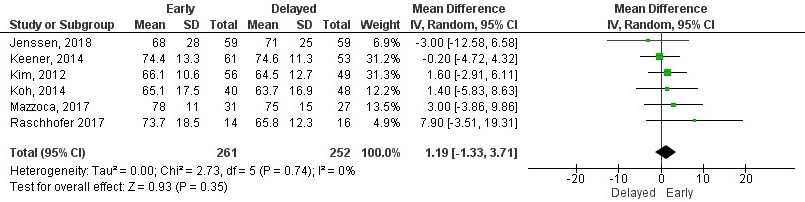


**Supplementary file 5.8**. Forest plot of function at six months by Constant-Murley score.


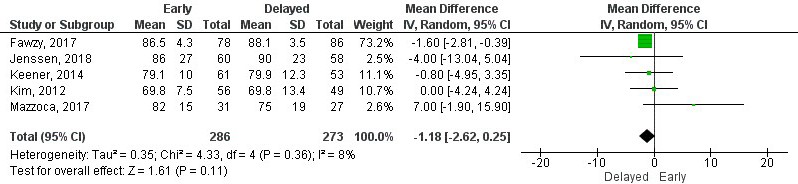
**Supplementary file 5.9**. Forest plot of function at one year by Constant-Murley score.


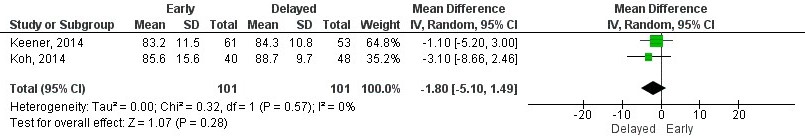


**Supplementary file 5.10**. Forest plot of function at two years by Constant-Murley score.


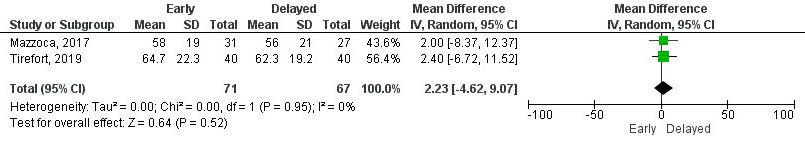
**Supplementary file 5.11**. Forest plot of function at three months by Single Assessment Numeric Evaluation.


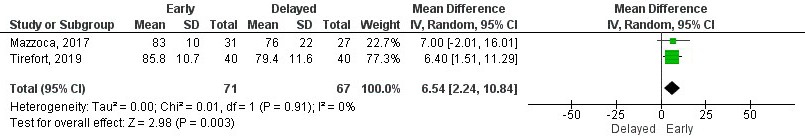


**Supplementary file 5.12**. Forest plot of function at six months by Single Assessment Numeric Evaluation.


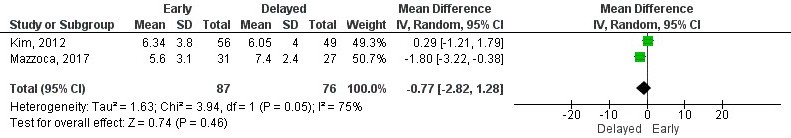
**Supplementary file 5.13**. Forest plot of function at three months by Simple Shoulder Test.


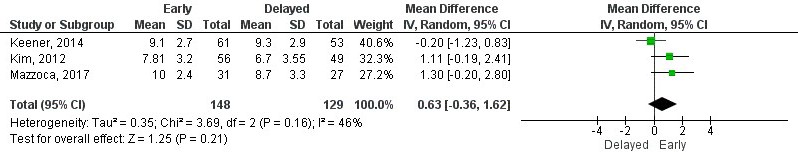


**Supplementary file 5.14**. Forest plot of function at six months by Simple Shoulder Test.


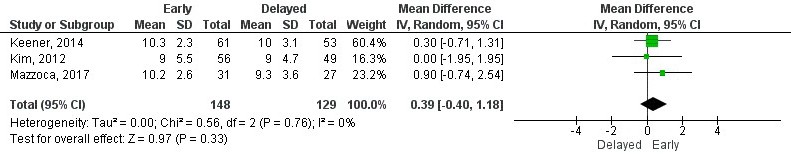
**Supplementary file 5.15**. Forest plot of function at one year by Simple Shoulder Test.


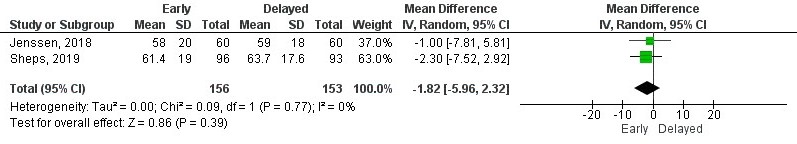


**Supplementary file 5.16.** Forest plot of function at three months by Western Ontario Rotator Cuff Index.


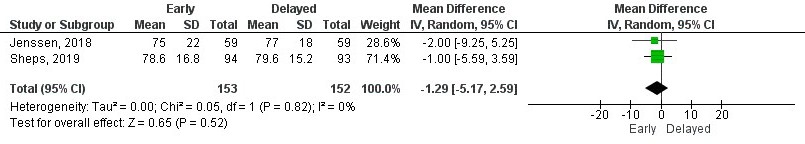
**Supplementary file 5.17.** Forest plot of function at six months by Western Ontario Rotator Cuff Index.


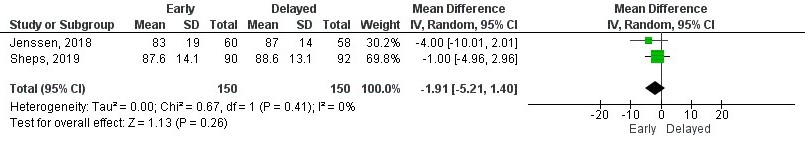


**Supplementary file 5.18.** Forest plot of function at one year by Western Ontario Rotator Cuff Index.


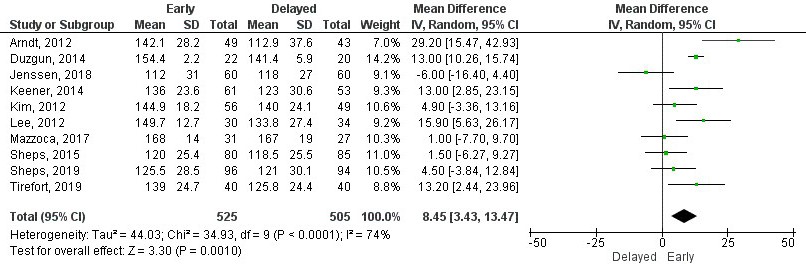

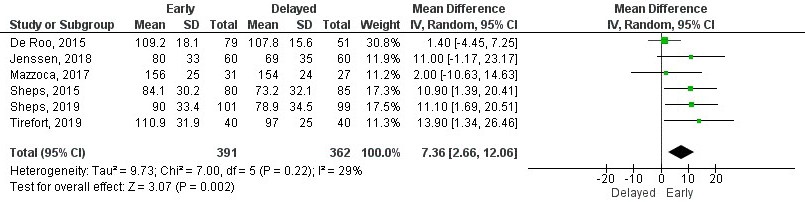
**Supplementary file 5.19.** Forest plot of range of movement for shoulder flexion at six weeks.

**Supplementary file 5.20.** Forest plot of range of movement for shoulder flexion at three months.


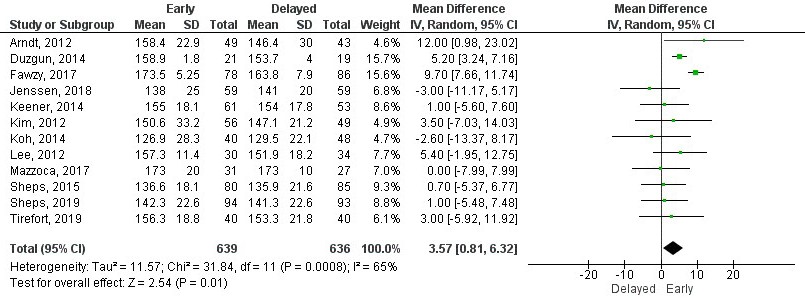
**Supplementary file 5.21.** Forest plot of range of movement for shoulder flexion at six months.


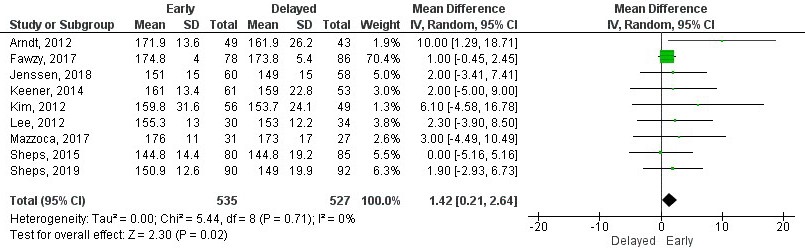


**Supplementary file 5.22.** Forest plot of range of movement for shoulder flexion at one year.


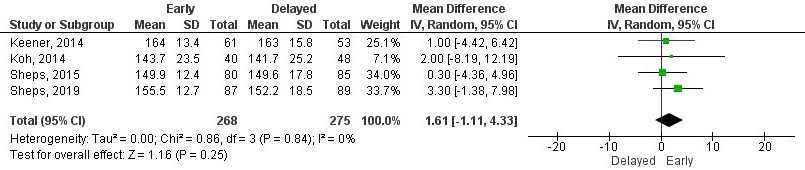
**Supplementary file 5.23.** Forest plot of range of movement for shoulder flexion at two years.


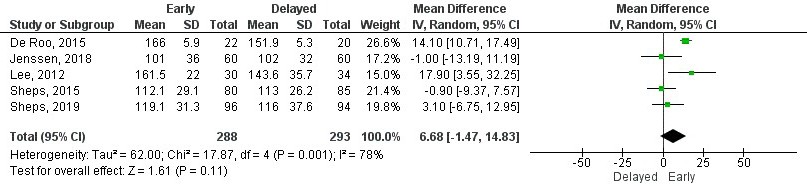

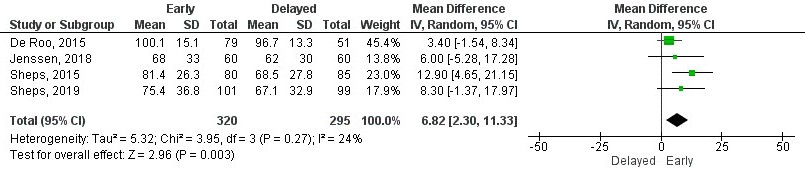
**Supplementary file 5.24.** Forest plot of range of movement for shoulder abduction at six weeks.

**Supplementary file 5.25.** Forest plot of range of movement for shoulder abduction at three months.


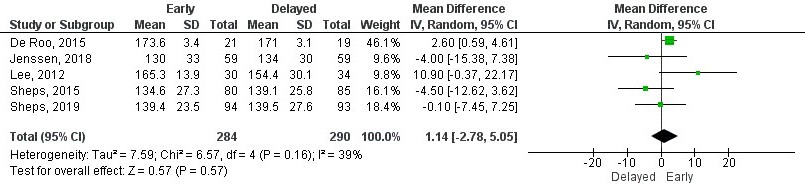
**Supplementary file 5.26.** Forest plot of range of movement for shoulder abduction at six months.


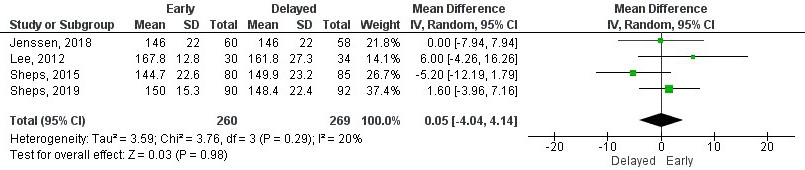


**Supplementary file 5.27.** Forest plot of range of movement for shoulder abduction at one year.


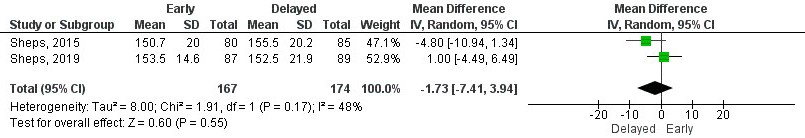


**Supplementary file 5.28.** Forest plot of range of movement for shoulder abduction at two years.


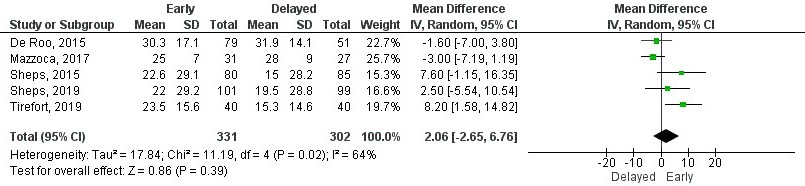


**Supplementary file 5.29.** Forest plot of range of movement for external rotation at six weeks.


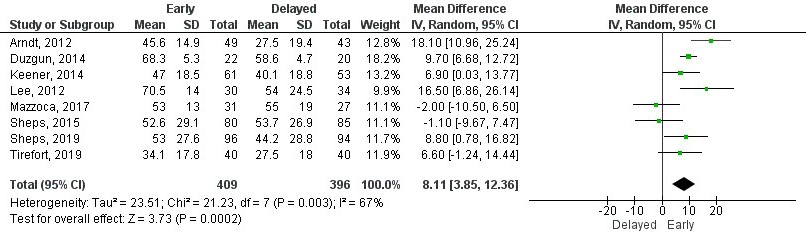
**Supplementary file 5.30.** Forest plot of range of movement for external rotation at three months.


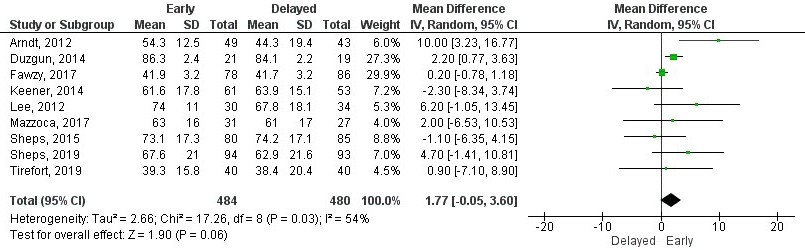


**Supplementary file 5.31.** Forest plot of range of movement for external rotation at six months.


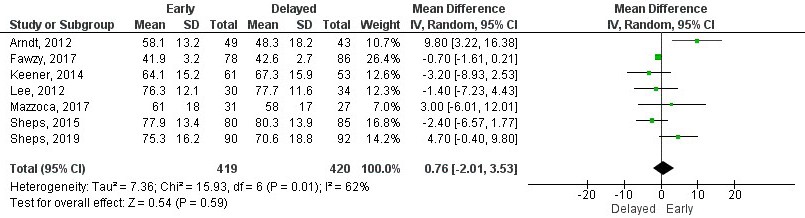
**Supplementary file 5.32.** Forest plot of range of movement for external rotation at one year.


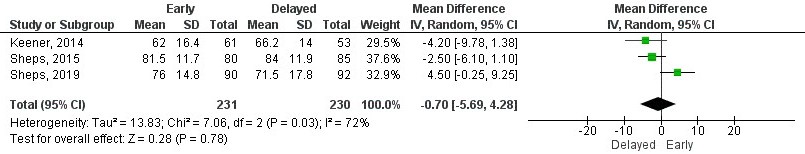


**Supplementary file 5.33.** Forest plot of range of movement for external rotation at two years.


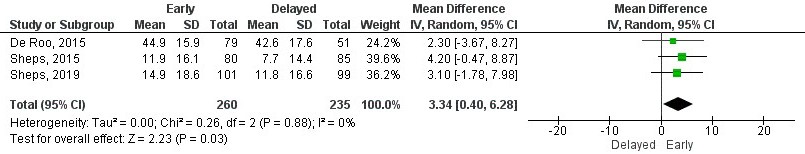
**Supplementary file 5.34.** Forest plot of range of movement for internal rotation at six weeks.


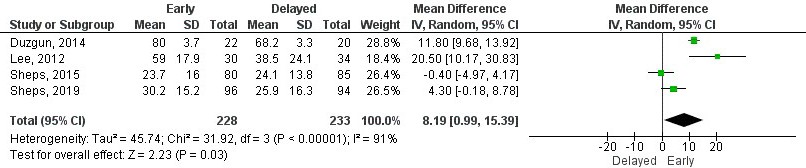


**Supplementary file 5.35.** Forest plot of range of movement for internal rotation at three months.


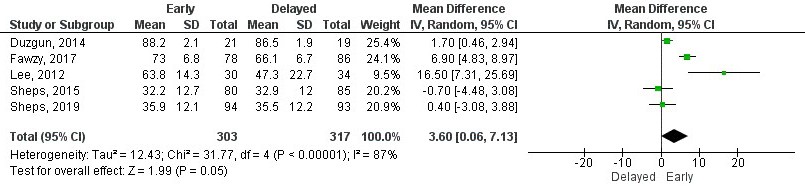


**Supplementary file 5.36.** Forest plot of range of movement for internal rotation at six months.


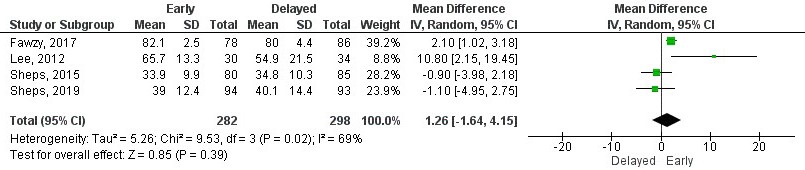


**Supplementary file 5.37.** Forest plot of range of movement for internal rotation at one year.


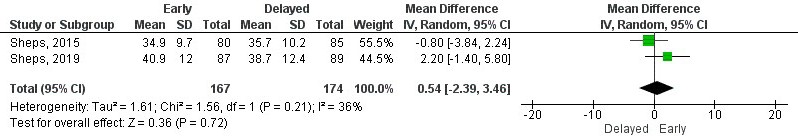


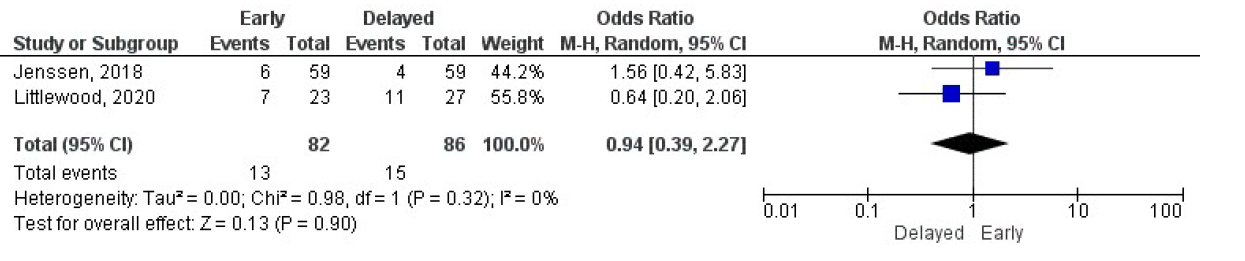
**Supplementary file 5.38.** Forest plot of range of movement for internal rotation at two years.

**Supplementary file 5.39.** Forest plot of odds ratio for repair integrity at three months.


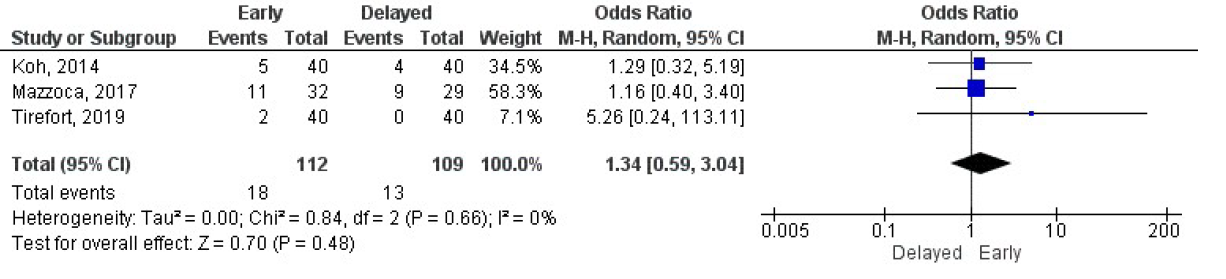


**Supplementary file 5.40.** Forest plot of odds ratio for repair integrity at six months.
