# Supplementary material for: Effectiveness of early versus delayed rehabilitation following rotator cuff repair: Systematic review and meta-analyses
Source: PLoS One. 2021 May 28;16(5):e0252137. doi: 10.1371/journal.pone.0252137 (PMC8162656; doi:10.1371/journal.pone.0252137)
Supplement: S6 File — (DOCX) [file pone.0252137.s006.docx]

**S6 File.** GRADE summary of findings.

| **Outcomes** | **№ of participants  (studies) Follow up** | **Certainty of the evidence (GRADE)** | **Relative effect (95% CI)** |
| --- | --- | --- | --- |
|  |  |  |  |
| Pain - Visual Analogue Scale at 6 weeks | 707 (6 RCTs) | ⨁⨁◯◯ LOW ^a,b^ | - |
| Pain - Visual Analogue Scale at 3 months | 692 (6 RCTs) | ⨁⨁⨁⨁ HIGH | - |
| Pain - Visual Analogue Scale at 6 months | 722 (7 RCTs) | ⨁⨁⨁⨁ HIGH | - |
| Pain - Visual Analogue Scale at 1 year | 521 (4 RCTs) | ⨁⨁⨁⨁ HIGH | - |
| Pain - Visual Analogue Scale at 2 years | 551 (4 RCTs) | ⨁⨁⨁⨁ HIGH | - |
| Function - American Shoulder and Elbow Score at 3 months | 243 (3 RCTs) | ⨁⨁⨁◯ MODERATE ^c^ | - |
| Function - American Shoulder and Elbow Score at 6 months | 365 (4 RCTs) | ⨁⨁⨁◯ MODERATE ^c^ | - |
| Function - American Shoulder and Elbow Score at 12 months | 441 (4 RCTs) | ⨁⨁◯◯ LOW ^d,e^ | - |
| Function - American Shoulder and Elbow Score at 24 months | 202 (2 RCTs) | ⨁⨁⨁◯ MODERATE ^c^ | - |
| Function - Constant-Murley score at 3 months | 313 (4 RCTs) | ⨁⨁◯◯ LOW ^c,e^ | - |
| Function - Constant-Murley score at 6 months | 513 (6 RCTs) | ⨁⨁⨁⨁ HIGH | - |
| Function Constant-Murley score at 12 months | 559 (5 RCTs) | ⨁⨁◯◯ LOW ^d,e^ | - |
| Function - Constant-Murley score at 24 months | 202 (2 RCTs) | ⨁⨁⨁◯ MODERATE ^c^ | - |
| Function - Single Assessment Numeric Evaluation at 3 months | 138 (2 RCTs) | ⨁⨁⨁◯ MODERATE ^c^ | - |
| Function - Single Assessment Numeric Evaluation at 6 months | 138 (2 RCTs) | ⨁⨁⨁◯ MODERATE ^c^ | - |
| Function - Simpe Shoulder Test at 3 months | 163 (2 RCTs) | ⨁◯◯◯ VERY LOW ^a,b,c,d^ | - |
| Function - Simple Shoulder Test at 6 months | 277 (3 RCTs) | ⨁⨁⨁◯ MODERATE ^c^ | - |
| Function - Simple Shoulder Test at 12 months | 277 (3 RCTs) | ⨁⨁⨁◯ MODERATE ^c^ | - |
| Function - Western Ontario Rotator Cuff Index at 3 months | 309 (2 RCTs) | ⨁⨁⨁◯ MODERATE ^c^ | - |
| Function - Western Ontario Rotator Cuff Index at 6 months | 363 (2 RCTs) | ⨁⨁⨁◯ MODERATE ^c^ | - |
| Function - Western Ontario Rotator Cuff Index at 12 months | 300 (2 RCTs) | ⨁⨁⨁◯ MODERATE ^c^ | - |
| Range of movement - Flexion at 6 weeks | 753 (6 RCTs) | ⨁⨁⨁⨁ HIGH | - |
| Range of movement - Flexion at 3 months | 1030 (10 RCTs) | ⨁⨁◯◯ LOW ^a,d^ | - |
| Range of movement - Flexion at 6 months | 1275 (12 RCTs) | ⨁⨁◯◯ LOW ^a,d^ | - |
| Range of Movement - Flexion at 12 months | 898 (9 RCTs) | ⨁⨁⨁◯ MODERATE ^d^ | - |
| Range of Movement - Flexion at 24 months | 543 (4 RCTs) | ⨁⨁⨁⨁ HIGH | - |
| Range of Movement - Abduction at 6 weeks | 615 (4 RCTs) | ⨁⨁⨁◯ MODERATE ^f^ | - |
| Range of Movement - Abduction at 3 months | 581 (5 RCTs) | ⨁⨁◯◯ LOW ^a,b,e,f^ | - |
| Range of Movement - Abduction at 6 months | 574 (5 RCTs) | ⨁⨁◯◯ LOW ^e,f^ | - |
| Range of Movement - Abduction at 12 months | 529 (4 RCTs) | ⨁⨁⨁◯ MODERATE ^e^ | - |
| Range of Movement - Abduction at 24 months | 341 (2 RCTs) | ⨁⨁⨁◯ MODERATE ^c^ | - |
| Range of Movement - External Rotation at 6 weeks | 633 (5 RCTs) | ⨁⨁⨁◯ MODERATE ^a^ | - |
| Range of Movement - External Rotation at 3 months | 805 (8 RCTs) | ⨁⨁⨁◯ MODERATE ^a^ | - |
| Range of Movement - External Rotation at 6 months | 964 (9 RCTs) | ⨁⨁◯◯ LOW ^a,d^ | - |
| Range of Movement - External Rotation at 12 months | 839 (7 RCTs) | ⨁⨁◯◯ LOW ^a,d^ | - |
| Range of Movement - External Rotation at 24 months | 461 (3 RCTs) | ⨁⨁⨁◯ MODERATE ^a^ | - |
| Range of Movement - Internal Rotation at 6 weeks | 495 (3 RCTs) | ⨁⨁⨁⨁ HIGH | - |
| Range of Movement - Internal Rotation at 3 months | 461 (4 RCTs) | ⨁◯◯◯ VERY LOW ^a,b,d,e^ | - |
| Range of Movement - Internal Rotation at 6 months | 620 (5 RCTs) | ⨁⨁◯◯ LOW ^a,d,e^ | - |
| Range of Movement - Internal Rotation at 12 months | 580 (4 RCTs) | ⨁⨁◯◯ LOW ^a,d,e^ | - |
| Range of Movement - Internal Rotation at 24 months | 341 (2 RCTs) | ⨁⨁⨁◯ MODERATE ^c^ | - |
| Repair integrity at 3 months | 168 (2 RCTs) | ⨁⨁⨁◯ MODERATE ^g^ | **OR 0.94** (0.39 to 2.27) |
| Repair integrity at 6 months | 221 (3 RCTs) | ⨁⨁⨁◯ MODERATE ^h^ | **OR 1.34** (0.59 to 3.04) |
| Repair integrity at 12 months | 960 (8 RCTs) | ⨁⨁◯◯ LOW ^d,i^ | **OR 1.26** (0.82 to 1.93) |

| ***The risk in the intervention group** (and its 95% confidence interval) is based on the assumed risk in the comparison group and the **relative effect** of the intervention (and its 95% CI).  **CI:** Confidence interval; **MD:** Mean difference; **OR:** Odds ratio  a. High heterogeneity  b. Large confidence interval. Confidence interval included both the point of no effect (0) and the minimal clinically important difference (MCID) for each outcome  MCID values:  - Pain: 1.4  - American Shoulder and Elbow Surgery: 11.1  - Constant Murley: 10.4  - Single Assessment Numeric Evaluation: 16.9  - Simple Shoulder Test: 2.3  - Western Ontario Rotator Cuff: 245  - Flexion range of movement: 14  - Abduction range of movement: 7  - Internal and external rotation range of momvent:15  c. Sample size <400 (200 per group)  d. A substantial number of trials were rated as of unclear and/or high risk of bias  e. Wide variance of point estimates across studies  f. Trial with a high risk of bias receiving most of the weight in the analysis  g. Small sample size (optimal information size: 8614 per group)  h. Small sample size (optimal information size: 1096 per group)  i. Small sample size (optimal information size: 3198 per group) |
| --- |
